# An update on *APC* I1307K homozygosity: observations from a large multigene panel testing cohort

**DOI:** 10.1007/s10689-026-00600-0

**Published:** 2026-09-17

**Authors:** Leah Zaretsky, Marcy E. Richardson, Taylor Coleman, Carrie Horton, Magan Trottier, Maggie Feng, Linda M. Polfus, Aimee L. Lucas

**Affiliations:** 1 Division of Medical Genetics and Genomics, Department of Genetics and Genomic Sciences, Icahn School of Medicine at Mount Sinai, New York, NY USA; 2 Ambry Genetics, Aliso Viejo, CA USA; 3 WCG Clinical Inc, Cary, NC USA; 4 Division of Gastroenterology and Hepatology, Mount Sinai Morningside and Mount Sinai West, Icahn School of Medicine at Mount Sinai, New York, NY USA

**Keywords:** *APC*, Homozygote, p.I1307K, p.Ile1307Lys, Colon cancer risk

## Abstract

Numerous studies have demonstrated that Ashkenazi Jewish individuals heterozygous for *APC* c.3902T > A; p.Ile1307Lys have 1.7-fold increased odds of colorectal adenocarcinoma (CRC). The cancer risk for homozygotes is unknown, and published data is based on small sample sizes. Here, we aim to quantitatively evaluate the odds of CRC in homozygotes from a clinical diagnostic setting. Analyses comparing the demographic and clinical histories among heterozygotes (*n* = 340) or homozygotes (*n* = 74) were compared to individuals with *MSH6*-associated Lynch syndrome (*n* = 372) and individuals from a pan-cancer panel of up to 90 genes who were negative for *APC* I1307K, *MSH6* (likely) pathogenic (LP/P) variants, and other reportable LP/P variants after exclusions, henceforth referred to as wildtype (WT, *n* = 19,809). Comparisons included Fisher’s exact or two-sample t-test and multivariate logistic regression. Homozygotes demonstrated a trend toward increased odds of CRC compared to WT (OR: 2.48; *p* = 0.074), and there was no difference between heterozygotes and WT (OR: 0.94; *p* = 0.83). *MSH6*-positive individuals had increased odds of CRC relative to homozygotes (OR: 3.54; *p* < 0.0001) but this was not significant in the logistic regression (OR: 1.19; *p* = 0.78). While homozygotes but not heterozygotes demonstrated an increase in CRC OR compared to WT, there was no statistically distinct odds of CRC between heterozygotes and homozygotes in this cohort. While the ORs herein support that the observed increase in CRC for homozygotes was more modest than that observed for *MSH6*-related Lynch syndrome in this cohort, current sample size and selection bias limit precise risk estimation and do not allow for distinction between modestly increased risk and no risk of CRC.

**Supplementary Information:**

The online version contains supplementary material available at https://doi.org/10.1007/s10689-026-00600-0.

## Introduction

Colorectal adenocarcinoma (CRC) is a leading cause of cancer-related morbidity and mortality worldwide [1]. Genetic predisposition plays a crucial role in the development of CRC, with several (likely) pathogenic (LP/P) variants identified as significant contributors to the onset of disease. One such variant, *APC* (NM_000038.6) c.3902T > A p.Ile1307K (I1307K), is known to generate an unstable tract of eight adenines which creates a hypermutable region of the gene and has been linked to an increased risk of CRC, albeit lower than other *APC* LP/P variants [2, 3]. Further research is needed to better understand the risks associated with *APC* I1307K [4], as the impact of this genetic variant in various populations, including its effect on homozygotes, remains inadequately explored [5].

The *APC* I1307K variant is found in the Ashkenazi Jewish (AJ) population with a frequency nearing 4% (gnomAD v.4.1.1). Several studies have indicated a lifetime CRC risk of 5–10% in *APC* I1307K heterozygotes in AJ populations [6, 7]. Other studies have similarly shown increases in CRC risk in smaller cohort and case series [2], further reinforcing the association between *APC* I1307K and increased CRC susceptibility. Based on these estimates of increased risk, the National Comprehensive Cancer Network (NCCN) currently recommends that *APC* I1307K heterozygotes receive CRC screening with colonoscopy every five years beginning at age 40 [8], in contrast to average-risk individuals, for whom colonoscopy is recommended every 10 years beginning at age 45 or 50, or for whom alternative screening strategies, including stool-based screening, may be used [9, 10]. The Hereditary Colorectal Cancer/Polyposis Variant Curation Expert Panel (VCEP), in conducting reviews of *APC* variants, stated that variant interpretation guideline criteria cannot be applied to frequent/low penetrant variants in *APC*, including I1307K, as the clinical presentation and disease mechanism of these variants are heterogeneous and complex [11]. The International Society for Gastrointestinal Hereditary Tumours (InSiGHT) currently does not recommend early screening in *APC* I1307K heterozygotes [4].

Despite the prevalence of the *APC* I1307K variant in individuals of AJ descent, the incidence of homozygotes is rare [6], and the mechanism of disease for this variant in this context remains poorly understood. The largest case series of homozygous individuals published to-date includes only four individuals, and these and other homozygous individuals reported in the literature exhibited wide phenotypic variability [12, 13].

In this report, a large cohort of individuals homozygous for *APC* I1307K was evaluated and compared to heterozygotes as well as positive (*MSH6+*) and negative (WT) control cohorts. The primary objective of this brief report is to quantitatively evaluate the CRC OR in *APC* I1307K homozygotes ascertained from a clinical diagnostic setting.

## Methods

### Study design and population

The primary objective of this study is to assess the OR of CRC in *APC* I1307K homozygotes. Additionally, we aim to describe the clinical characteristics and phenotypic expression of *APC* I1307K homozygous individuals. In this retrospective cohort study, de-identified data from individuals who underwent multigene panel testing for hereditary cancer indications were evaluated. Individuals underwent a multigene panel test (up to 90 genes) [14] between May 2012 and April 2024 at a single diagnostic testing laboratory (Ambry Genetics, Aliso Viejo, CA). Statistical models used WT and *MSH6*-positives as negative and positive control cohorts, respectively. For *APC* I1307K homozygotes, all available cases were clinically curated. For *APC* I1307K heterozygotes, *MSH6*-positives, and WT cohorts, only select individuals had been curated for a variety of reasons including billing purposes, resulting in curation that is nearly random. There was a small proportion of individuals who were curated based on genotype- and/or phenotype-related projects; however, these are a minority of cases and are expected to be diverse enough so as not to strongly sway the data.

Individuals heterozygous for LP/P variants in *MSH6* were included as a comparison group, given this gene’s well-established cancer risks and management recommendations, which are considered to be at the relatively lower end of the spectrum among Lynch syndrome susceptibility genes. *MSH6* heterozygotes are generally recommended to have colonoscopies every one to three years beginning at age 30 to 35 due to a cumulative risk for CRC through age 70 of around 12% [15].

The WGC IRB (formerly the Western Institutional Review Board) determined that the study was exempt from the Office for Human Research Protection Regulations for the Protection of Human Subjects (45 CFR 46) and provided a waiver of consent for Ambry de-identified data analyzed collectively.

Clinical characteristics were obtained from clinician-completed test requisition forms and supplemented with clinical documentation, including pedigrees and clinical notes, when available. The clinical and demographic characteristics included age at testing, age at diagnosis (if available), sex at birth, self-reported ethnicity, immunohistochemistry (IHC) status, and microsatellite instability levels. Reportable variants included variants of uncertain significance (VUS) and LP/P variants as determined by laboratory-specific guidelines modified from the American College of Medical Genetics and Genomics (ACMG) and the Association for Molecular Pathology (AMP) [16]. Patients were excluded from analyses if they had co-occurring LP/P variants in any gene on the ordered cancer panel. Co-occurring VUSs were allowed for all cohorts, as most VUSs that are reclassified are downgraded to benign. The WT cohort was negative for *APC* I1307K, *MSH6* LP/P variants, and other reportable LP/P variants after exclusions. Additional exclusion criteria for the *APC* I1307K heterozygote/homozygote and WT cohorts included those with abnormal IHC for *PMS2*, *MSH2*, *MLH1*, or *MSH6* or patients with abnormal MSI (“MSI-High”) supporting a Lynch- or Lynch-like tumor unassociated with *APC*.

### Statistical analysis

Power calculations were performed under a beta of 20%, an alpha of 0.05, and a possible effect size similar to literature-established risks for *APC* I1307K heterozygotes (OR = 1.5–1.7). Differences between groups were compared using Fisher’s exact test for cohort comparisons and two-sample t-test for differences in age at diagnosis. Unadjusted and adjusted odds ratios (OR) were calculated for clinical characteristics using univariate logistic regression and multivariable logistic regression adjusting for age, sex, and ethnicity. Statistical analyses were performed across three ethnicity categories: all ethnicities combined; participants self-identifying as AJ; and participants self-identifying as AJ or White, combined. To maximize the number of *APC* I1307K homozygotes and heterozygotes and to preserve the *MSH6* comparison group, which had only 10 individuals self-reporting AJ ethnicity, we elected to present the all-ethnicities model. ORs for CRC with 95% confidence intervals (CI) were summarized by two-way comparisons of genotype. All statistical tests were conducted using R v.4.3.0 with a p-value of less than 0.05 considered statistically significant.

## Results

Clinical and demographic counts for the *APC* I1307K homozygote (*n* = 74), *APC* I1307K heterozygote (*n* = 340), *MSH6*-positive (*n* = 372), and WT (*n* = 19,809) cohorts are shown in Table 1. Of the 74 homozygous individuals, 56 (75.7%) reported AJ ancestry, 5 (6.8%) had a history of CRC, and 1 (1.4%) had a history of more than one primary CRC. The heterozygous cohort included 141 (41.5%) individuals with reported AJ ancestry, 13 (3.8%) with a history of CRC, and 1 (0.3%) with a history of more than one primary CRC. A smaller proportion of the *MSH6*-positive cohort reported AJ ancestry (*n* = 10, 2.7%), while 76 (20.4%) had a history of CRC and 7 (1.9%) had a history of more than one CRC. The WT cohort included 593 (3.0%) individuals with reported AJ ancestry, 885 (4.5%) with a history of any number of CRC, and 23 (0.1%) with a history of more than one primary CRC.

**Table 1:** Patient clinical history and demographics

	APC I1307K		Positive controls	Negative controls
	Homozygous (*n* = 74)	Heterozygous (*n* = 340)	*MSH6*+ (*n* = 372)	WT (*n* = 19,809)
*Sex*				
Female	59 (79.7)	270 (79.4)	277 (74.5)	16,599 (83.8)
Male	15 (20.3)	70 (20.6)	95 (25.5)	3209 (16.2)
Unknown	0	0	0	1 (< 0.1)
*Ethnicity*				
White	8 (10.8)	123 (36.2)	199 (53.5)	10,376 (52.4)
African American/Black	0	10 (2.9)	79 (21.2)	1715 (8.7)
Hispanic	0	5 (1.5)	8 (2.2)	1016 (5.1)
Ashkenazi Jewish	56 (75.7)	141 (41.5)	10 (2.7)	593 (3.0)
Asian	2 (2.7)	3 (0.9)	12 (3.2)	689 (3.5)
Mixed ethnicity	2 (2.7)	2 (0.6)	4 (1.1)	251 (1.3)
Middle Eastern	2 (2.7)	7 (2.1)	1 (0.3)	75 (0.4)
Native American	0	0	0	29 (0.1)
Pacific Islander	0	0	0	2 (< 0.1)
Alaskan Native	0	0	0	1 (< 0.1)
Other	0	3 (0.9)	3 (0.8)	157 (0.8)
Unknown	4 (5.4)	46 (13.5)	56 (15.1)	4905 (24.8)
*Current age*				
N	74	340	371	19,809
Mean (SD)	60.4 (13.2)	53.6 (14.7)	52.9 (13.3)	51.9 (13.7)
Range (min, max)	34, 92	18, 92	17, 87	6, 95
Quartiles (25th, median, 75th )	52, 61, 69	43, 54, 64	43, 54, 62	42, 52, 62
*Any cancer diagnosis*				
Yes	47 (63.5)	149 (43.8)	224 (60.2)	10,661 (53.8)
No	27 (36.5)	180 (52.9)	139 (37.4)	8582 (43.3)
Not provided	0	11 (3.2)	9 (2.4)	566 (2.9)
*Age at any cancer diagnosis*				
N	44	125	198	10,064
Mean (SD)	59.0 (13.0)	54.1 (12.7)	53.1 (11.6)	53.4 (13.5)
Range (min, max)	37, 91	5, 92	17, 87	1, 95
Quartiles (25th, median, 75th )	49, 57, 68	47, 54, 62	45, 53, 60	44, 53, 63
*CRC diagnosis*				
Any number of CRC	5 (6.8)	13 (3.8)	76 (20.4)	885 (4.5)
> 1 primary	1 (1.4)	1 (0.3)	7 (1.9)	23 (0.1)
No CRC	69 (93.2)	327 (96.2)	296 (79.6)	18,924 (95.5)
*Age at first CRC diagnosis*				
N	5	13	60	863
Mean (SD)	52.5 (5.7)	52.1 (13.0)	51.2 (11.7)	52.5 (12.5)
Range (min, max)	45, 59	26, 72	17, 81	12, 95
Quartiles (25th, median, 75th )	49, 55, 55	45, 55, 58	44, 50, 59	45, 50, 60
*Polyps*				
Yes	15 (20.3)	60 (17.6)	63 (16.9)	2575 (13.0)
No	12 (16.2)	36 (10.6)	42 (11.3)	1662 (8.4)
Not provided	47 (63.5)	244 (71.8)	267 (71.8)	15,572 (78.6)

**Fig. 1:**
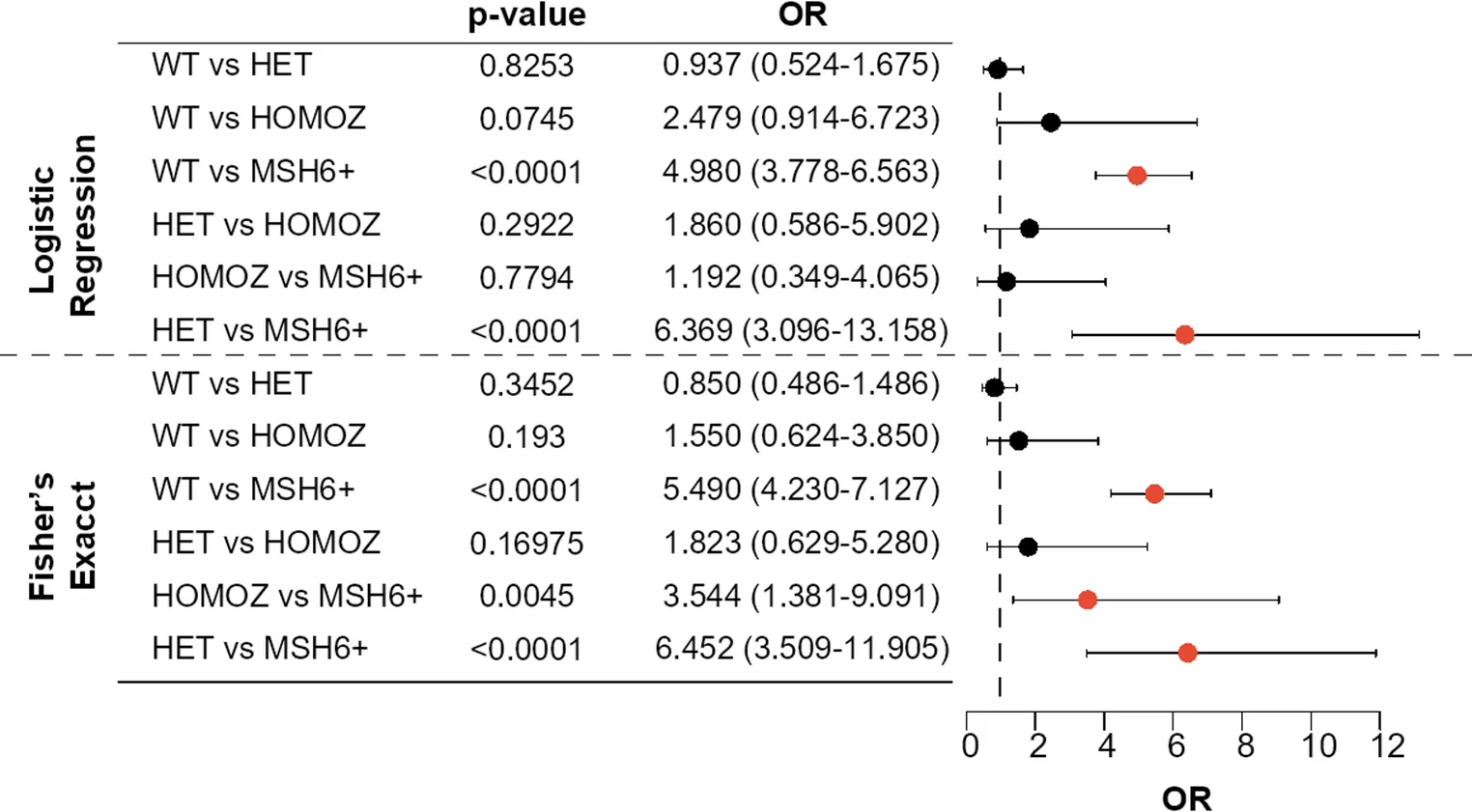
Forest plots depicting the odds ratios (ORs) and 95% confidence intervals for both logistic regression (above dotted line) and Fisher’s exact test (below dotted line) for comparisons of CRC OR among cohorts. The reference cohort is listed first in the pair in column 1. Statistically significant differences (*p* < 0.05) are represented by red dots, and non-significant (N/S) differences are represented by black dots. Vertical dashed line represents an OR of 1

On univariable analysis, *MSH6*-positive individuals demonstrated a higher OR for CRC compared to WT (OR: 5.49; 95% CI 4.23 to 7.13; *p* < 0.0001), heterozygous (OR: 6.45; 95% CI 3.51 to 11.91; *p* < 0.0001), and homozygous groups (OR: 3.54: 95% CI 1.38 to 9.10; *p* = 0.005). Similar results were found on multivariable analysis. All other logistic regression comparisons were non-significant. Although non-significant, a comparison of *APC* I1307K homozygotes and WT trended toward significance in the logistic regression model (OR: 2.48; 95% CI 0.91 to 6.72; *p* = 0.075) (Fig. 1).

Although underpowered, sensitivity analysis limited to AJ individuals was performed using the same analysis methods. The comparisons of CRC between cohorts using the Fisher’s exact test and the cohort covariate for all logistic regression models were not statistically significant. No *MSH6*-positive individuals in the AJ subpopulation had CRC so results for these comparisons are not evaluable (Supplemental Table 1).

**Fig. 2:**
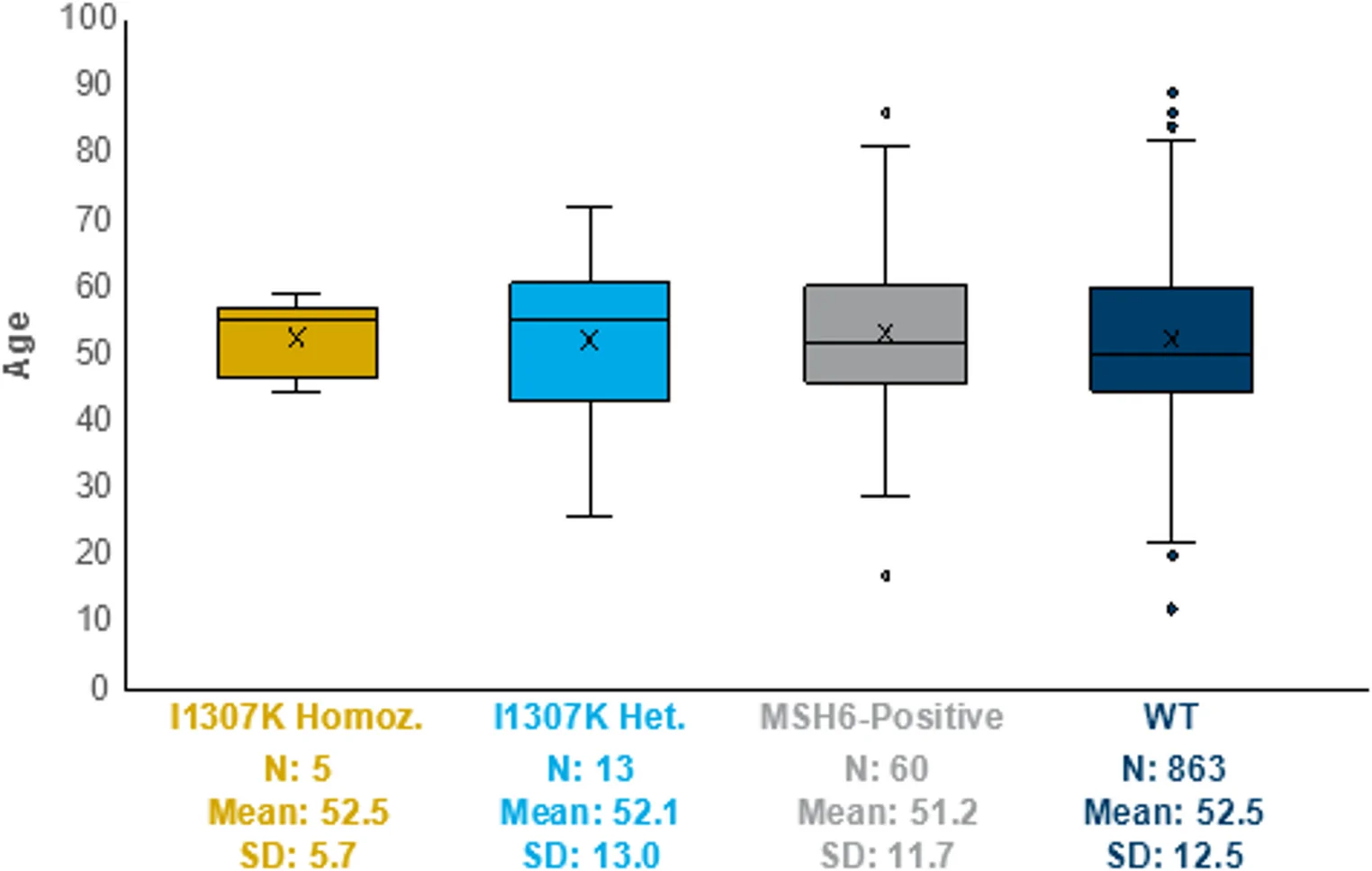
Box and whisker plot depicting the median and interquartile range for age at first CRC onset (y-axis) for each cohort. The number of individuals with a CRC diagnosis who reported the age at onset is provided under the x-axis. Not all individuals with CRC reported the age at which they were diagnosed

Unlike previous case control studies, there were no differences in the OR for CRC in WT versus *APC* I1307K heterozygotes in this clinical cohort (OR: 0.85; 95% CI 0.49 to 1.49; *p* = 0.35). Additionally, there was no significant difference in age at the first CRC diagnosis between cohorts (Fig. 2).

To determine the detectable OR with the current sample size, limited by homozygotes with *n* = 74, power calculations were performed under a model with alpha set to 0.05. When comparing homozygotes and heterozygotes, there is 80% power to detect an OR of 3.8. Similarly, for comparisons between homozygotes and WT, there is 80% power to detect an OR between 3 and 3.2. However, considering that homozygotes may have ORs on the order of those described in the literature for heterozygotes (OR 1.5–1.7), the minimum number of homozygotes needed to detect these effect sizes with 80% power at alpha of 0.05 is *n* = 567 for a comparison with heterozygotes (OR 1.78) and *n* = 723 for a comparison with WT (OR 1.54).

Power calculations for the sample sizes needed to detect an OR of variable size comparing Homozygotes to Heterozygotes or Wildtype. With the current homozygous sample size (*n* = 74), there is approximately 80% power to detect an OR of 3.0 when compared to Wildtype and an approximate OR of 3.8 when compared to Heterozygotes.

## Discussion

Counseling *APC* I1307K homozygotes on CRC risk and medical management is challenging, given the limited size and scope of current case reports. Given the molecular mechanism of *APC* I1307K whereby a hypermutable region of the gene is created, questions remain regarding the impact of carrying copies of the variant on both alleles, as is the case for homozygotes, and whether the CRC risk is further increased as compared to individuals carrying only one copy of the variant. Homozygosity for other LP/P variants in the *APC* gene have not been reported and are expected to be embryonic lethal. In this study, we describe a large cohort of homozygotes and aim to quantitatively evaluate the associated CRC OR.

A strength of this analysis includes the reporting of the largest cohort of homozygous *APC* I1307K carriers to date. Since publicly available population-based cohorts still have small numbers of homozygotes, a large clinical cohort was necessary to detect small effect sizes; however, the inherent selection bias may also obscure these differences. The *APC* I1307K homozygote cohort in our study trends toward increased odds of CRC compared to WT. Although the estimated OR did not reach significance for a number of analyses and confidence intervals were wide, these findings add to the limited evidence available for *APC* I1307K homozygosity and provide additional data to inform future risk estimates. Given the wide confidence intervals, these results should be interpreted with caution and should not be viewed as sufficient to support changes to clinical management. Rather, they should be considered in the context of existing literature and underscore the need for additional studies with larger sample sizes.

Small effect sizes continue to limit the ability to detect significantly distinct odds of CRC between *APC* I1307K homozygotes and heterozygotes, with 567 homozygotes needed to detect an OR for CRC similar to previously published ORs for heterozygotes. Further data would be needed to confirm true ORs, as lack of power and selection bias limit these inferences.

This study has several important limitations. First, this clinical cohort is not representative of the general population, as all individuals, including those in the negative control group, underwent multigene panel testing for hereditary cancer based on personal and/or family history, resulting in selection bias. However, the vast majority of this cohort is ascertained for breast cancer predisposition, and thus, it is expected that the selection bias, particularly in the negative control cohort, will not strongly influence colorectal cancer analyses. Nevertheless, selection bias and under-reporting of ancestry likely underlie why we were unable to replicate the small effect size (OR 1.7) previously described in the literature, even after adjusting for age, sex, and ethnicity. Second, although the curated dataset is of high quality, full clinical data and pathology reports were not available and/or documented in detail for every individual in the control cohorts (I1307K heterozygous, WT, and *MSH6*-positive). As a result, cancer diagnoses and polyp data may be underreported. Polyp data was exploratory and incomplete, as polyp information was not provided for most individuals included in the study. Additionally, only 5 homozygotes in this cohort had CRC, resulting in an OR estimate that is unstable and limits conclusions regarding magnitude of risk. As a result, our study cannot distinguish between modestly increased risk and no risk of CRC. Finally, ancestry data for the cohort were self-reported and, despite *APC* I1307K being a known founder variant in this population, not all individuals with this variant reported AJ ancestry.

In conclusion, in our study cohort, *APC* I1307K homozygosity demonstrated a trend toward increased OR of CRC compared with WT; however, the estimated OR was more modest than that observed for *MSH6*-related Lynch syndrome in this cohort. While these findings suggest a potential association, the current sample size limits precise risk estimation. Larger, well-powered studies are needed to better define the magnitude of CRC risk associated with *APC* I1307K homozygosity and to clarify its implications for clinical management and guideline development.

## Electronic Supplementary Material

Below is the link to the electronic supplementary material.

Supplementary Material 1

## Data Availability

Ambry Genetics supports open data sharing. All data generated or analyzed during this study are included in this published article and its supplementary files, excepting detailed patient data that would compromise HIPAA-compliant safeguards applied to protect patient privacy.

## References

[1] Siegel RL, Wagle NS, Cercek A, Smith RA, Jemal A (2023) Colorectal cancer statistics. CA Cancer J Clin 73:233–254. 10.3322/caac.21772 36856579

[2] Zauber NP, Sabbath-Solitare M, Marotta S, Zauber AG, Foulkes W, Chan M et al (2005) Clinical and genetic findings in an Ashkenazi Jewish population with colorectal neoplasms. Cancer 104:719–729. 10.1002/cncr.21230 15959913

[3] Syngal S, Schrag D, Falchuk M, Tung N, Farraye FA, Chung D et al (2000) Phenotypic characteristics associated with the *APC* gene I1307K mutation in Ashkenazi Jewish patients with colorectal polyps. JAMA 284:857–860. 10.1001/jama.284.7.857 10938175

[4] Valle L, Katz LH, Latchford A, Mur P, Moreno V, Frayling IM et al (2023) Position statement of the international society for gastrointestinal hereditary tumours (InSiGHT) on *APC* I1307K and cancer risk. J Med Genet 60:1035–1043. 10.1136/jmg-2022-108984 PMC10646901 37076288

[5] Allen A, Rowlands CF, Latchford A, Turnbull C, Valle L (2025) *APC* I1307K and clinical management: insights from UK Biobank association analysis of colorectal and other cancer risks in Ashkenazi and non-Ashkenazi whites. J Med Genet 62:767–771. 10.1136/jmg-2025-110911 PMC12703324 40866199

[6] Woodage T, King SM, Wacholder S, Hartge P, Struewing JP, McAdams M et al (1998) The *APC* I1307K allele and cancer risk in a community-based study of Ashkenazi Jews. Nat Genet 20:62–65. 10.1038/1722 9731533

[7] Boursi B, Sella T, Liberman E, Shapira S, David M, Kazanov D et al (2013) The *APC* p.I1307K polymorphism is a significant risk factor for CRC in average risk Ashkenazi Jews. Eur J Cancer 49:3680–3685. 10.1016/j.ejca.2013.06.040 23896379

[8] National Comprehensive Cancer Network NCCN Guidelines: genetic/familial high-risk assessment: colorectal, endometrial, esophageal, and gastric [Internet]. Version 1.2026 [cited 2026 June 28]. Available from: https://www.nccn.org/professionals/physician_gls/pdf/genetics_ceeg.pdf

[9] US Preventive Services Task Force (2021) Screening for colorectal cancer: US preventive services task force recommendation statement. JAMA 325:1965–1977. 10.1001/jama.2021.6238 34003218

[10] Wolf AMD, Fontham ETH, Church TR, Flowers CR, Guerra CE, LaMonte SJ et al (2018) Colorectal cancer screening for average-risk adults: 2018 guideline update from the American Cancer Society. CA: A Cancer J Clin. 68:250–281. 10.3322/caac.21457 29846947

[11] Spier I, Yin X, Richardson M, Pineda M, Laner A, Ritter D et al (2023) Gene-specific ACMG/AMP classification criteria for germline *APC* variants: recommendations from the ClinGen InSiGHT hereditary colorectal cancer/polyposis variant curation expert panel. Genet Med 26(2):100992. 10.1016/j.gim.2023.100992 PMC10922469 37800450

[12] Rosenblum A, Springer M, Eppolito A, Axell L, Mohler L (2021) Homozygous Germline *APC* p.I1307K variants: a case series. Case Rep Oncol 14:1295–1303. 10.1159/000518683 PMC8460922 34720931

[13] Zauber P, Sabbath-Solitare M, Stephen PM, Chamberlain R, Chong G, Foulkes WD et al (2018) Sporadic desmoid tumor in an Ashkenazi patient homozygous for the *APC*I1307K* gene mutation. Acta Oncol 47:1158–1161. 10.1080/02841860701716900 18770064

[14] CancerNext-Expanded [Internet]. California: Ambry Genetics [cited 2026 January 25]. Available from: https://www.ambrygen.com/providers/genetic-testing/28/oncology/cancernext-expanded

[15] Wang C, Wang Y, Hughes KS, Parmigiani G, Braun D (2020) Penetrance of colorectal cancer among mismatch repair gene mutation carriers: a meta-analysis. JNCI Cancer Spectr 4(5):pkaa027. 10.1093/jncics/pkaa027 PMC7476651 32923933

[16] LaDuca H, Polley EC, Yussuf A, Hoang L, Gutierrez S, Hart SN et al (2020) A clinical guide to hereditary cancer panel testing: evaluation of gene-specific cancer associations and sensitivity of genetic testing criteria in a cohort of 165,000 high-risk patients. Genet Med 22:407–415. 10.1038/s41436-019-0633-8 PMC7000322 31406321

