# Supplementary material for: An update on APC I1307K homozygosity: observations from a large multigene panel testing cohort
Source: Fam Cancer. 2026 Sep 17;25(4):89. doi: 10.1007/s10689-026-00600-0 (PMC13585870; doi:10.1007/s10689-026-00600-0)
Supplement: Supplementary file 1 [file 10689_2026_600_MOESM1_ESM.docx]

**Supplemental Table 1** Patient Clinical History and Demographics [Ashkenazi Jewish]

|  | ***APC* I1307K** | | ***Positive Controls*** | **Negative Controls** |
| --- | --- | --- | --- | --- |
|  | **Homozygous (*n* = 56)** | **Heterozygous (*n* = 141)** | ***MSH6*+ (*n* = 10)** | **WT (*n* = 593)** |
| **Sex** | | | | |
| Female | 46 (82.1) | 115 (81.6) | 9 (90.0) | 482 (81.3) |
| Male | 10 (17.9) | 26 (18.4) | 1 (10.0) | 111 (18.7) |
| **Current Age** | | | | |
| N | 56 | 141 | 10 | 593 |
| Mean (SD) | 63.1 (12.6) | 54.5 (14.3) | 47.7 (11.9) | 49.8 (13.4) |
| Range (min, max) | 36, 92 | 22, 92 | 30, 65 | 20, 89 |
| Quartiles (25th , median, 75th ) | 55, 64, 71 | 44, 53, 64 | 40, 47, 59 | 39, 50, 60 |
| **Any Cancer Diagnosis** | | | | |
| Yes | 37 (66.1) | 63 (44.7) | 3 (30.0) | 232 (39.1) |
| No | 19 (33.9) | 75 (53.2) | 6 (60.0) | 343 (57.8) |
| Not Provided | 0 | 3 (2.1) | 1 (10.0) | 18 (3.0) |
| **Age at Any Cancer Diagnosis** | | | | |
| N | 35 | 53 | 3 | 222 |
| Mean (SD) | 61.4 (12.7) | 53.3 (14.9) | 48.0 (15.7) | 52.2 (13.7) |
| Range (min, max) | 44, 91 | 5, 92 | 34, 65 | 1, 89 |
| Quartiles (25th , median, 75th ) | 50, 59, 71 | 46, 55, 62 | 34, 45, 65 | 43, 52, 62 |
| **CRC Diagnosis** | | | | |
| Any number of CRC | 2 (3.6) | 6 (4.3) | 0 | 12 (2.0) |
| > 1 primary | 0 | 0 | 0 | 0 |
| No CRC | 54 (96.4) | 135 (95.7) | 10 (100.0) | 581 (98.0) |
| **Age at First CRC Diagnosis** | | | | |
| N | 2 | 6 | 0 | 11 |
| Mean (SD) | 46.8 (3.2) | 49.4 (13.7) |  | 50.4 (8.4) |
| Range (min, max) | 45, 49 | 26, 64 |  | 35, 65 |
| Quartiles (25th , median, 75th ) | 45, 47, 49 | 41, 54, 58 |  | 45, 51, 56 |
| **Polyps** | | | | |
| Yes | 13 (23.2) | 25 (17.7) | 0 | 16 (11.1) |
| No | 10 (17.9) | 17 (12.1) | 0 | 65 (11.0) |
| Not Provided | 33 (58.9) | 99 (70.2) | 10 (100.0) | 462 (77.9) |

**Supplemental Table 2** Required Sample Size for Comparing *APC* I1307K Homozygotes with Heterozygotes and Wildtype

| **Power 80%, alpha 0.05**  **Required Sample Sizes** | **Detectable OR** | | | | | | | | | | | | | | |
| --- | --- | --- | --- | --- | --- | --- | --- | --- | --- | --- | --- | --- | --- | --- | --- |
|  | **1.55** | **1.67** | **1.78** | **1.82** | **2.00** | **2.20** | **2.40** | **2.60** | **2.80** | **3.00** | **3.20** | **3.40** | **3.60** | **3.80** | **4.00** |
|  | | | | | | | | | | | | | | | |
| **Homozygote** | nd | nd | 567 | 517 | 369 | 271 | 210 | 169 | 140 | 119 | 103 | 90 | 80 | 72 | 65 |
| **Heterozygote** | nd | nd | 2636 | 2403 | 1715 | 1260 | 976 | 786 | 651 | 553 | 479 | 418 | 372 | 335 | 302 |
|  | | | | | | | | | | | | | | | |
| **Homozygote** | 723 | 533 | nd | 365 | 246 | 179 | 137 | 110 | 90 | 76 | 65 | 57 | 50 | nd | nd |
| **Wildtype** | 193539 | 142686 | nd | 97707 | 65852 | 47916 | 36673 | 29446 | 24092 | 20344 | 17400 | 15258 | 13384 | nd | nd |

nd = not done
